# All-trans retinoic acid combined with the VAH regimen for EVI1-positive acute myeloid leukemia: a case report with a brief literature review

**DOI:** 10.3389/fonc.2026.1790087

**Published:** 2026-04-15

**Authors:** Yingxin Zhao, Hongxia Wang, Jiaxin Lv, Pei Pei, Zhengyang Zhang, Bei Zhang, Zhixin Pei, Junjun Bai, Jingjing Gu, Huimin Wu, Fei Wang, Yi Zhang, Qinglin Song

**Affiliations:** 1 Department of Hematology, Jiaozuo People’s Hospital, Jiaozuo, Henan, China; 2 Laboratory of Hematological Diseases, Jiaozuo People’s Hospital, Jiaozuo, Henan, China; 3 Department of Rehabilitation, Jiaozuo People’s Hospital, Jiaozuo, Henan, China; 4 Department of Clinical Pharmacy, Jiaozuo People’s Hospital, Jiaozuo, Henan, China

**Keywords:** acute myeloid leukemia, all-trans retinoic acid, EVI1, refractory leukemia, VAH regimen

## Abstract

EVI1 positivity is a well-recognized high-risk factor in acute myeloid leukemia (AML), typically characterized by complex molecular genetic abnormalities, suboptimal responses to conventional chemotherapy, and a poor prognosis. We report a case of a 42-year-old patient with EVI1-positive AML harboring the MLL-AF6 fusion gene, who failed to achieve remission after undergoing standard “IA” induction therapy and was then treated with VAH (venetoclax, azacitidine, and homoharringtonine) consolidation chemotherapy. The patient subsequently received all-trans retinoic acid (ATRA) (20 mg twice daily, administered orally) and achieved complete remission with incomplete hematologic recovery (CRh) after 24 days, accompanied by a marked reduction in EVI1 expression relative to baseline levels. Following this, consolidation therapy consisting of one cycle of “VAH plus ATRA” led to complete remission (CR). This case suggests that the combination of ATRA with the VAH regimen may demonstrate promising efficacy and an acceptable safety profile in patients with EVI1-positive AML who are refractory to conventional chemotherapy. However, further clinical studies are required to confirm its wider applicability.

## Introduction

Acute myeloid leukemia (AML) is a highly heterogeneous malignant hematologic disorder. Among its subtypes, AML with EVI1 overexpression accounts for approximately 8%-10% of all cases and represents a high-risk category characterized by distinct clinicobiological features and extremely poor prognosis (1–3). Previous research has demonstrated that patients with EVI1-positive AML exhibit a markedly inferior response to conventional chemotherapy (4). To date, no clearly effective targeted therapy has been established for this subtype, posing considerable challenges in clinical management.

All-trans retinoic acid (ATRA), a classical differentiation-inducing agent that facilitates the maturation of leukemic cells along the granulocytic lineage (5, 6). Recent preclinical studies have indicated its potential therapeutic efficacy in non-APL AML (7). Preclinical evidence suggests that ATRA combined with chemotherapy may enhance the elimination of EVI1-positive leukemic cells and diminish minimal residual disease (MRD) within the bone marrow (8–10). These findings suggest a promising therapeutic avenue for this challenging high-risk AML subtype.

To further explore the potential clinical utility of ATRA in EVI1-positive AML, we present the diagnostic and therapeutic course of a patient treated with ATRA combined with the VAH regimen. We also provide a focused narrative review of key clinical evidence supporting ATRA’s potential in this high-risk subtype. This case review the potential mechanistic rationale, offering preliminary clinical insights and a novel therapeutic perspective for this patient population.

## Case presentation

**Figure 1:**
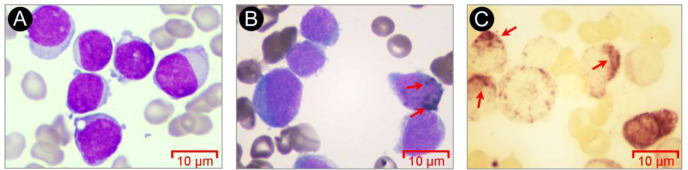
Morphological analysis and staining with POX and α-NAE in bone marrow cells at the initial stage of diagnosis. **(A)** Morphological examination of bone marrow cells via Giemsa staining. **(B)** POX staining of bone marrow cells. **(C)** α-NAE staining of bone marrow cells. Images collected at 1000×, scale bar = 10 μm.

**Figure 2:**
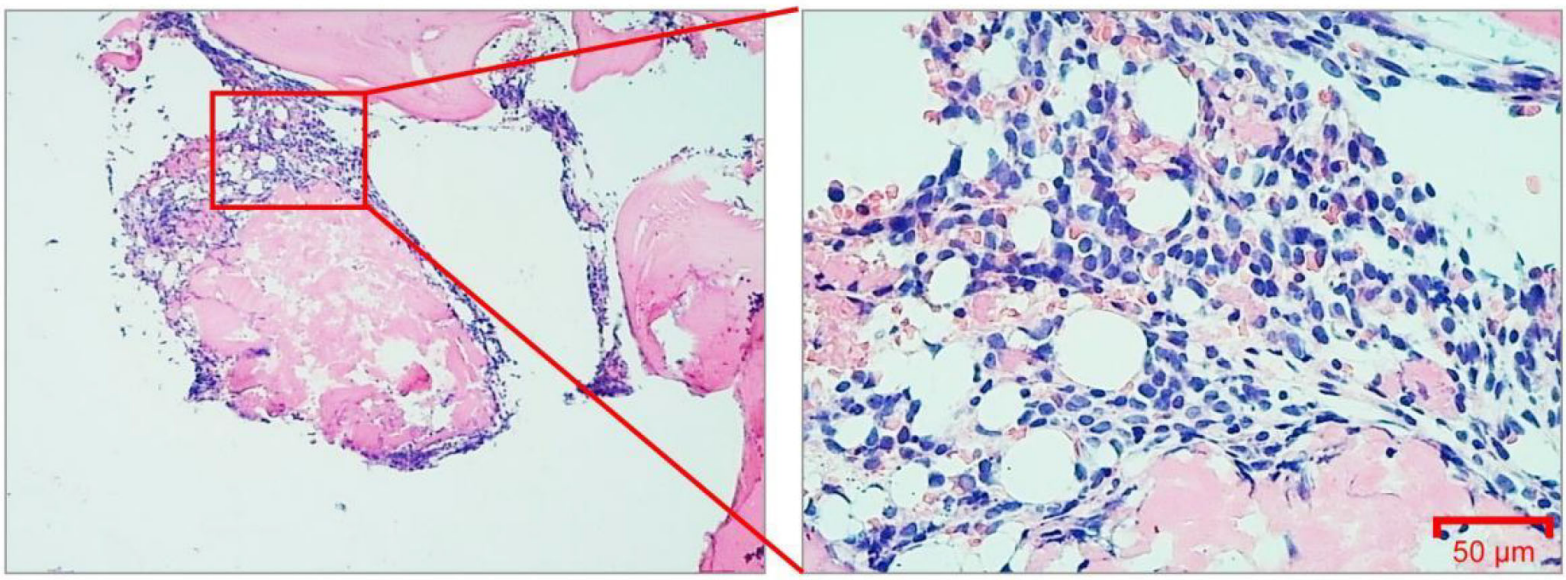
Histological examination of bone marrow via H&E staining. H&E staining of bone marrow. Images collected at 400×, scale bar = 50 μm.

In May 2025, a 42-year-old male patient was admitted to our hospital with fever. The complete blood count (CBC) showed a white blood cell (WBC) count of 7.44×10^9^/L, hemoglobin (HB) level of 82.00 g/L, platelet (PLT) count of 29.00×10^9^/L, and an absolute neutrophil count (NEU) of 0.03×10^9^/L (**Table 1**). Bone marrow cytology revealed markedly hypercellular marrow and blasts accounting for 87.0% of nucleated cells (**Figure 1A**). Bone marrow cells exhibited a positive reaction to the POX stain (**Figure 1B**), and the α-NAE stain yielded positive results that were inhibitable by NaF (**Figure 1C**). Bone marrow biopsy revealed hematopoietic area accounting for approximately 40% and prolymphocyte proportion reaching about 85% (**Figure 2**).

**Table 1:** Summary of the white blood cell count, hemoglobin level, platelet count and absolute neutrophil count in peripheral blood at different stages.

Date	Regimen	WBC (×10^9^/L)	HB (g/L)	PLT (×10^9^/L)	NEU (×10^9^/L)	Response status
5.13	Diagnosis	7.44	82	29	0.03	
5.20-5.26	IA					
6.6	–	15.52	73	45	0.05	NR
6.12-6.18	VAH					
6.24	Venetoclax	0.38	62	41	0.01	
6.24-7.02	Venetoclax+ATRA					
7.17	–	1.41	75	80	1.01	CRh
8.18-8.26	VAH+ATRA					
9.24	–	1.23	99	74	0.54	CR
10.21	HSCT					CR

WBC, white blood cell; HB, hemoglobin; PLT, platelet; NEU, neutrophil; NR, no remission; CRh, complete remission with incomplete hematologic recovery;CR, complete remission.

**Figure 3:**
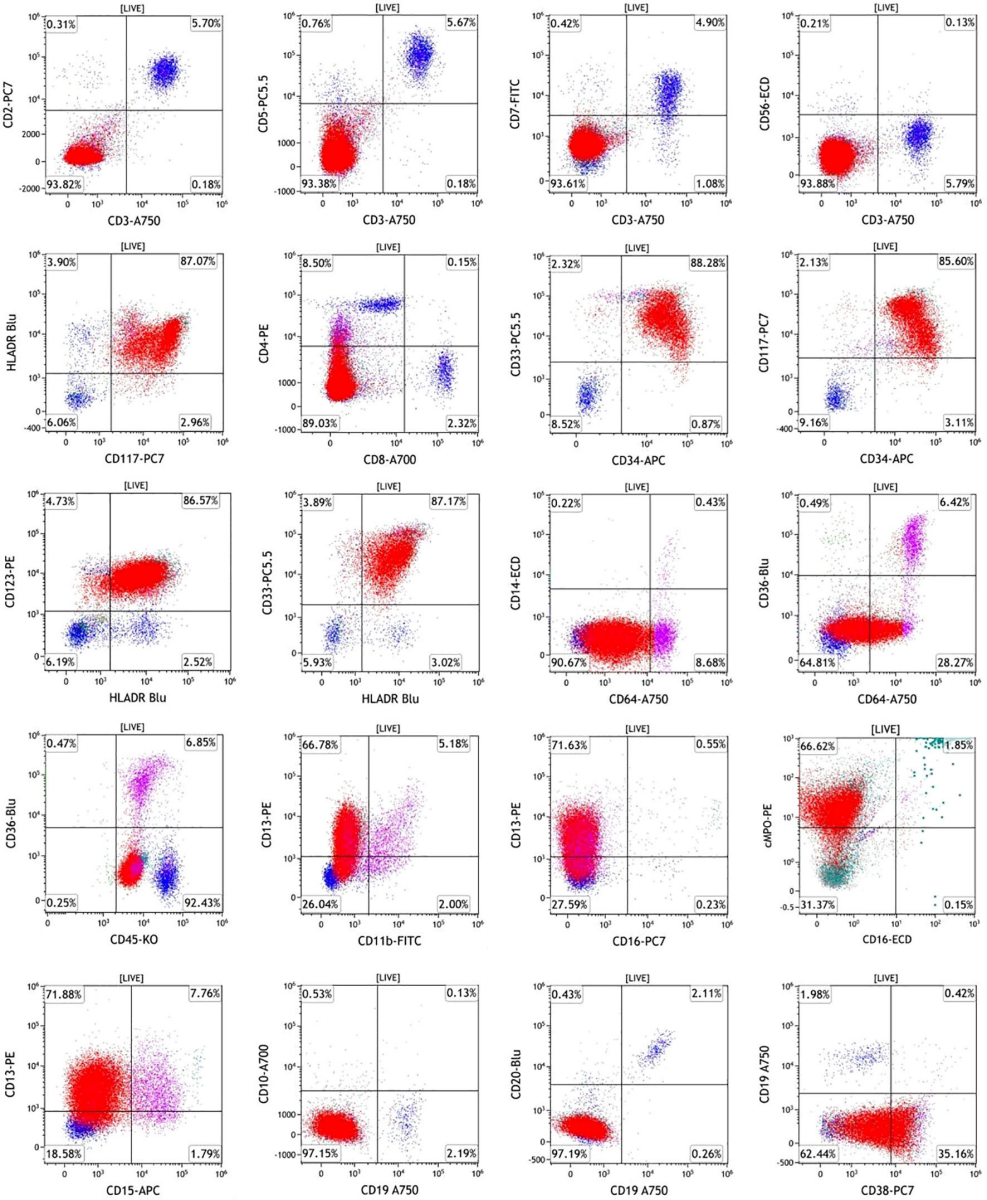
Flow cytometry analysis of a bone marrow aspirate at the initial stage of diagnosis. Bone marrow flow cytometry showing acute leukemia diagnosis: 85.60% blasts/immature myeloid cells expressed CD34^+^, CD117^+^, HLA-DR^+^, CD123^+^, CD33^+^, CD13^+^, partial CD38^+^, minor CD64^+^, and cMPO^+^, lacking expression of CD56, CD19, CD10, CD3, CD7, CD5, CD2, and CD14. 6.42% of the monocytes expressed CD33^+^, HLA-DR^+^, CD13^+^, CD64^+^, CD36^+^, CD11b^+^, CD15^+^, CD4^+^, and minimal CD14^+^ expression, lacking expression of CD34 and CD117.

Flow cytometric analysis of the bone marrow revealed an aberrant immunophenotype indicative of acute leukemia, characterized by 85.60% blasts/immature myeloid cells. Furthermore, approximately 6.42% of the monocytes exhibited an immunophenotype characterized (**Figure 3**).

Cytogenetic analysis revealed a normal male karyotype: 46, XY (20). Whole-genome copy number alteration (CNA) profiling demonstrated 46, XY karyotype with no evidence of copy-neutral loss of heterozygosity (CN-LOH) in critical regions. Molecular assays revealed WT1 expression at 74.15% and EVI1 expression at 136.25%. Screening for fusion transcripts identified the presence of the MLL-AF6 fusion. Next-generation sequencing (NGS) identified a TTN p.R25309H missense mutation with a variant allele frequency of 49.70%, which was a secondary mutation and had a weak correlation with the disease, with no pathogenic mutations detected in tier I or III genes. Integrating morphological, immunophenotypic, and molecular findings, the final diagnosis was acute myeloid leukemia with MLL-AF6 fusion and high EVI1 expression, categorized as an adverse-risk subtype.

The patient initiated “IA” induction chemotherapy on May 20, 2025 (cytarabine 100 mg·m^-^²·day^-^¹ for 7 days; idarubicin 10 mg·m^-^²·day^-^¹ on days 1-3). On day 11 post-treatment initiation, peripheral blood smear revealed 42% blasts, indicating no remission (NR). Although menin inhibitor therapy was recommended due to the presence of MLL rearrangement, the patient declined this option owing to financial constraints.

**Figure 4:**
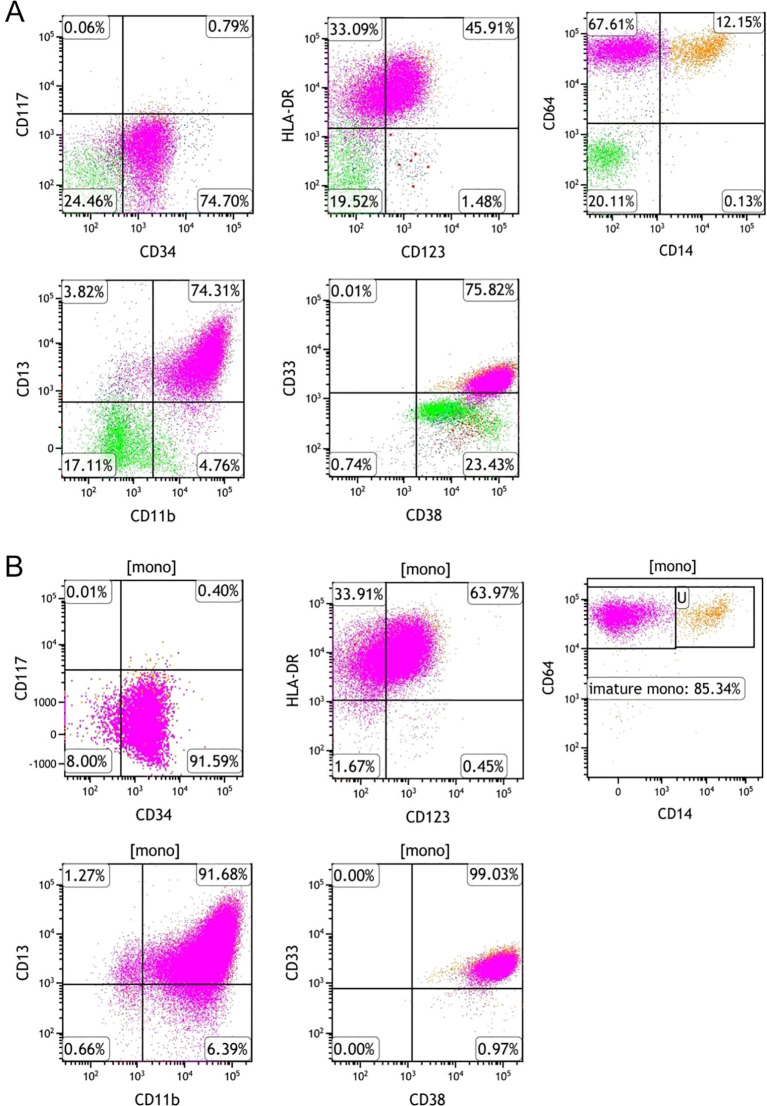
Flow cytometry analysis of bone marrow aspirate after VAH regimen (before ATRA addition). **(A)** Myeloblasts (CD34^+^CD117^+^) were markedly reduced to 0.79% following VAH treatment, indicating effective clearance of the leukemic blast population. **(B)** A large population of immature monocytes (85.34% of monocytic cells) persisted, exhibiting an immunophenotype of CD64^+^, CD13^+^, CD33^+^, CD123^+^, HLA−DR^+^, CD38^+^, with dim expression of CD34 and CD11b. This residual immature monocytic component represented persistent leukemia-related cells requiring further differentiation therapy.

Treatment was subsequently adjusted to the “VAH” regimen: venetoclax (100 mg on day 1, 200 mg on day 2, then 400 mg daily on days 4-21, orally), azacitidine(75 mg·m^-^²·day^-^¹ for 7 days), and homoharringtonine(2 mg·day^-^¹ for 7 days). On day 12 of chemotherapy, bone marrow examination demonstrated hypocellular marrow with diminished granulopoiesis, increased monocytic cells, and 38% blast-like monocytes. Flow cytometry revealed a marked reduction in myeloid blasts (CD34^+^CD117^+^) to 0.79%, with no aberrant immunophenotype detected (**Figure 4A**). Within the monocytic cell population, 85.34% were identified as immature monocytes (**Figure 4B**). CBC at this time showed WBC 0.38×10^9^/L, HB 62.00 g/L, PLT 41.00×10^9^/L, and NEU 0.01×10^9^/L (**Table 1**).

**Figure 5:**
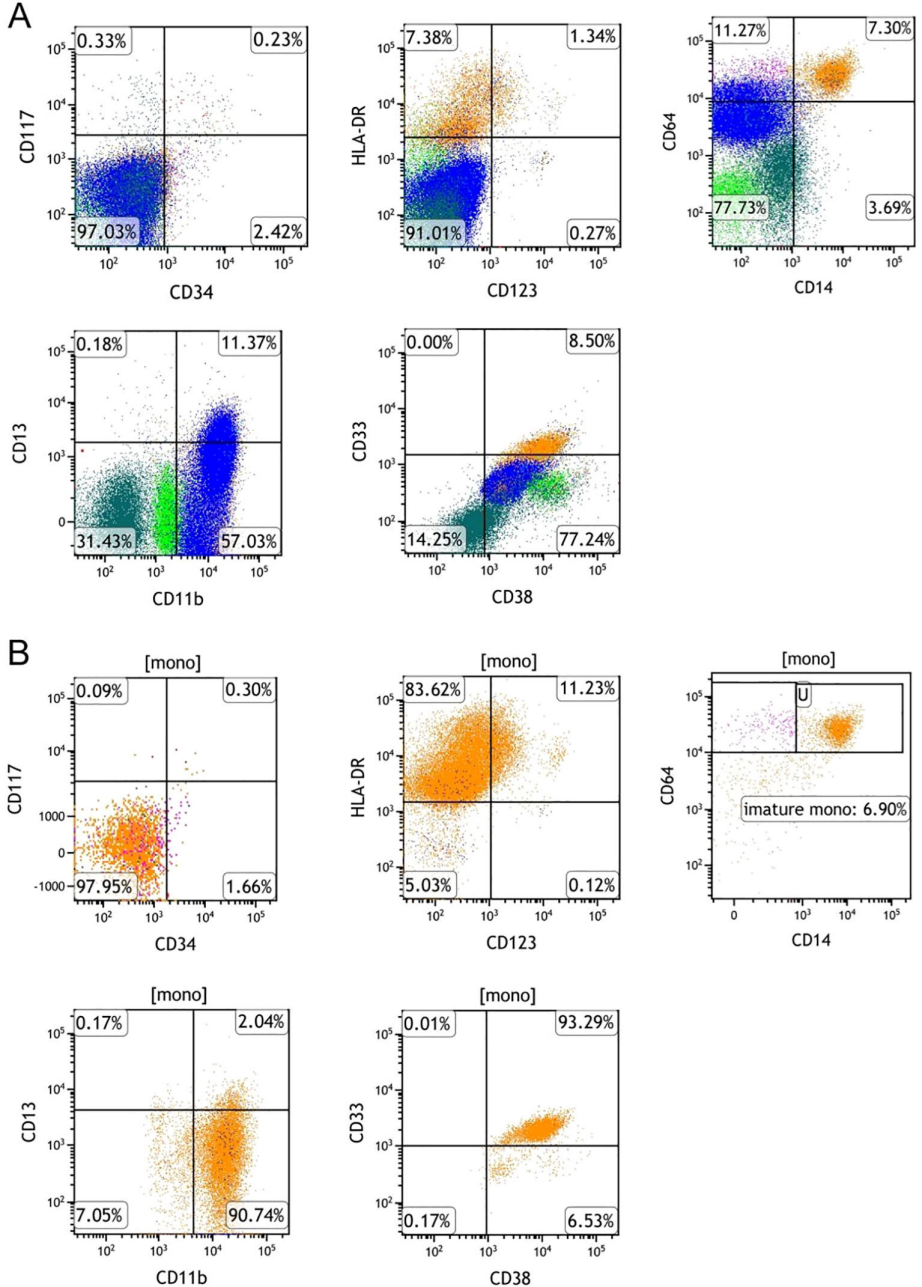
Flow cytometry analysis of bone marrow aspirate after addition of ATRA to the VAH regimen. **(A)** Myeloblasts remained at a low level (0.23%), and no aberrant immunophenotype was detected. **(B)** The proportion of immature monocytes decreased to 6.90%, with no immunophenotypic abnormalities, and normal myelopoiesis recovered.

Given the patient’s EVI1 overexpression, all-trans retinoic acid (ATRA; 20 mg twice daily) was added to the ongoing venetoclax-based regimen. On day 15 following completion of therapy, CBC indicated hematopoietic recovery, WBC 1.41×10^9^/L, HB 75 g/L, PLT 80×10^9^/L, NEU 1.01×10^9^/L (**Table 1**). Bone marrow aspiration revealed active hematopoiesis without detectable blasts or immature cells. Minimal residual disease (MRD) assessment by flow cytometry revealed a myeloblast population of 0.23% and an immature monocyte proportion of 6.90%, with no immunophenotypic abnormalities (**Figures 5A, B**). Molecular analysis demonstrated the persistence of the MLL-AF6 fusion transcript at 0.69% and a reduction of the EVI1 expression to 0.46%. Based on morphological, immunophenotypic, and molecular findings, the patient achieved complete remission with incomplete hematologic recovery (CRh).

The patient subsequently received one cycle of consolidation therapy with the “ATRA + VAH” regimen. Follow-up evaluation confirmed sustained CR (**Table 2**). The patient has since successfully undergone haploidentical hematopoietic stem cell transplantation with his son as the donor. So far, the patient remains in CR, without graft-versus-host disease (GVHD) or non-relapse mortality (NRM).

**Table 2:** Summary of the marrow assessment and gene quantification at different stages.

Date	Regimen	Peripheral blood assessment	Marrow Assessment	EVI1 levels (%)	MLL-AF6 levels (%)	Response status
5.13	Diagnosis	75.00% immature myeloid blasts	85.60% immature myeloid blasts	136.25	positive	–
5.20-5.26	IA	–	–	–	–	–
6.6	–	42.00% immature myeloid blasts	–	–	–	NR
6.12-6.18	VAH	–	–	–	–	–
6.24	Venetoclax	0% immature myeloid blasts	0.79% immature myeloid blasts and 85.34% immature monocytes	–	–	–
6.24-7.02	Venetoclax+ATRA	–	–	–	–	–
7.17	–	0% immature myeloid blasts	0.23% immature myeloid blasts and 6.90% immature monocyte	0.46	0.69	CRh
8.18-8.26	VAH +ATRA	–	–	–	–	–
9.24	–	0% immature myeloid blasts	0% immature myeloid blasts	–	–	CR
10.21	HSCT					CR

Treatment response was assessed according to the ELN Guidelines Version 2022 for Acute Myeloid Leukemia (Age ≥18 Years) (11). Complete remission (CR) was defined as morphologic CR with bone marrow blasts <5%, absolute neutrophil count (ANC) >1,000/mcL, platelets ≥100,000/mcL, and transfusion independence. The term CR with partial hematologic recovery (CRh) has been introduced for patients with morphologic bone marrow blast clearance and partial recovery of both neutrophils (≥0.5 *10^9^/L [500/uL]) and platelets (≥50 *10^9^/L [50 000/uL]). No response (NR) was defined as patients evaluable for response but not meeting the criteria for CR, CRh, CRi, MLFS, or PR.

## Discussion

The ecotropic viral integration site 1 (EVI1) gene, located at chromosome 3q26, encodes a zinc-finger transcription factor essential for hematopoietic stem cell self-renewal and is critically involved in leukemic cell proliferation, differentiation, and apoptosis (12). EVI1-positive acute myeloid leukemia (AML) is frequently associated with recurrent genetic abnormalities, including 11q23 (MLL rearrangement), inv(3)(q21q26)/t(3;3)(q21;q26), and monosomy 7. Co-occurring mutations in FLT3, C-KIT, along with a high incidence of MLL rearrangements, contribute to its adverse molecular profile (13–17). Emerging evidence indicates that EVI1 is a critical transcriptional target of MLL oncoproteins in hematopoietic stem cells, while fusion proteins such as MLL-AF9 sustain aberrant overexpression of EVI1 (18). These interactions establish a synergistic pathogenic network that drives leukemogenesis and may contribute to therapeutic resistance (19).

Management of EVI1-positive AML remains a major clinical challenge. Standard induction regimens such as the conventional protocol (anthracycline plus cytarabine) or other high-intensity chemotherapy, provide limited efficacy in this patient subgroup (10). While allogeneic hematopoietic stem cell transplantation can significantly improve long-term survival, achieving a higher rate of complete remission prior to transplantation remains a critical and unmet clinical need.

Mechanistically, EVI1 acts as a key transcriptional regulator shared by hematopoietic stem cells and leukemic stem cells. Its overexpression disrupts normal myeloid differentiation by interacting with transcription factors such as GATA-1, thereby sustaining the self-renewal capacity of leukemic stem cells (20). Notably, all-trans retinoic acid (ATRA) modulates EVI1 expression in both leukemic cell lines and primary AML samples, while EVI1 itself modulates retinoic acid signaling pathway and enhances the antileukemic effects of ATRA (21). *In vitro* studies by Verhagen et al. demonstrated that ATRA induces differentiation and apoptosis in EVI1-positive AML cells; the combination of ATRA and doxorubicin resulted in significantly reduced cell viability compared to doxorubicin alone, suggesting that ATRA may enhance remission rates in this high-risk AML subgroup (10).

Furthermore, downregulation of EVI1 triggers extensive epigenetic reprogramming (16, 22). The combination of decitabine and ATRA exerts synergistic cytotoxicity in elderly patients with AML via modulation of the miR-34a/MYCN axis, providing a mechanistic rationale for integrating ATRA with hypomethylating agents in EVI1-high AML (23). A retrospective analysis of 13 high-risk AML patients with EVI1 overexpression treated with ATRA-containing regimens revealed encouraging response rates (24). Notably, ATRA combined with azacitidine produced high remission rates, and patients achieving complete remission (CR) demonstrated survival exceeding one year, highlighting its potential therapeutic value. However, larger studies are warranted for confirmation (24).

Emerging evidence also shows that ATRA can overcome venetoclax resistance by downregulating the expression of BCL-2 and MCL-1. The combination of venetoclax, azacitidine, and ATRA has been associated with high rates of composite complete remission in newly diagnosed AML, and the differentiation-promoting properties of ATRA may facilitate platelet recovery and mitigate hematologic toxicity (24, 25).

Homoharringtonine (HHT) effectively targets leukemia stem-like cells and exhibits significant activity when combined with hypomethylating agents, such as azacitidine, in elderly and high-risk AML (22, 23). The combination of HHT with venetoclax further inhibits the MAPK/ERK and PI3K/AKT signaling pathways, producing enhanced antileukemic synergy (26–28).

Based on these mechanistic and clinical insights, the patient’s treatment strategy was transitioned to the VAH regimen (venetoclax, azacitidine, and homoharringtonine) following failure of IA induction. The VAH regimen cleared the majority of myeloid blasts, but left a residual population of 85.32% immature monocytes. Subsequent induction of differentiation with all-trans retinoic acid (ATRA) ultimately led to complete remission (CR), accompanied by a reduction in the quantitative levels of both EVI1 and MLL-AF6 genes. Following haploidentical hematopoietic stem cell transplantation, the patient remains in CR to date, without GVHD or NRM.

Despite the encouraging response, this study has several limitations inherent to a single-case report. The findings are derived from a single-patient observation and therefore lack statistical power and generalizability; thus, larger cohort studies are required to validate the therapeutic effect. Additionally, long-term follow-up is essential to determine the durability of remission and its impact on progression-free survival. Moreover, the molecular mechanisms underlying EVI1 downregulation, including specific epigenetic regulatory pathways remain incompletely understood. In addition, no functional assays were performed in this patient to directly interrogate ATRA induced differentiation pathways. Future research will involve systematic collection of multicenter clinical cases alongside parallel mechanistic investigations to more comprehensively evaluate the efficacy and biological underpinnings of this therapeutic strategy.

## Conclusion

In summary, the pathogenesis of EVI1-positive AML is highly complex and frequently associated with multiple high-risk genetic abnormalities, suggesting that a singular, uniform treatment approach is improbable. ATRA, through its ability to induce differentiation and promote apoptosis, may enhance the eradication of EVI1-positive leukemic cells when integrated into combination regimens. Treatment regimens incorporating ATRA, such as the ATRA plus VAH combination employed in this case, may represent a promising therapeutic avenue. Future multicenter clinical trials are warranted not only to validate the efficacy and safety of this regimen but also to optimize the combination strategy (e.g.treatment regimen and optimal dosing). This study aims to provide more individualized treatment basis for such refractory AML patients.

## Data Availability

The raw data supporting the conclusions of this article will be made available by the authors, without undue reservation.
